# The protective action of osmolytes on the deleterious effects of gamma rays and atmospheric pressure plasma on protein conformational changes

**DOI:** 10.1038/s41598-017-08643-1

**Published:** 2017-08-18

**Authors:** Pankaj Attri, Minsup Kim, Thapanut Sarinont, Eun Ha Choi, Hyunwoong Seo, Art E. Cho, Kazunori Koga, Masaharu Shiratani

**Affiliations:** 1 Plasma Bioscience Research Center/Department of Electrical and Biological Physics, Kwangwoon University, Seoul, 01897 Korea; 2 Faculty of Information Science and Electrical Engineering, Kyushu University, Fukuoka, Japan; 3 Department of Bioinformatics, Korea University, Sejong, 02841 Korea; 4 Graduate School of Information Science and Electrical Engineering, Kyushu University, Fukuoka, Japan

## Abstract

Both gamma rays and atmospheric pressure plasma are known to have anticancer properties. While their mechanism actions are still not clear, in some contexts they work in similar manner, while in other contexts they work differently. So to understand these relationships, we have studied Myoglobin protein after the treatment of gamma rays and dielectric barrier discharge (DBD) plasma, and analyzed the changes in thermodynamic properties and changes in the secondary structure of protein after both treatments. The thermodynamic properties were analyzed using chemical and thermal denaturation after both treatments. We have also studied the action of gamma rays and DBD plasma on myoglobin in the presence of osmolytes, such as sorbitol and trehalose. For deep understanding of the action of gamma rays and DBD plasma, we have analyzed the reactive species generated by them in buffer at all treatment conditions. Finally, we have used molecular dynamic simulation to understand the hydrogen peroxide action on myoglobin with or without osmolytes, to gain deeper insight into how the osmolytes can protect the protein structure from the reactive species generated by gamma rays and DBD plasma.

## Introduction

Cancer is a complex disease that holds the ability to metastasize into different organs of the body, which results in the increase of cancer. Cancer cases worldwide are predicted to almost double in the next 20–40 years^1, 2^. A huge proportion of cancer patients suffer deterioration for years or spans later^3, 4^ causing a therapeutic challenge. For the clinical management of cancer, radiation remains by far the most utilized treatment for patients with localized malignant tumors, and it is applied in a course of multiple fractions for several weeks to decrease the toxicity in normal cell^5–7^. Low and high linear energy transfer (LET) radiations are using for radiation therapy to efficiently kill the tumor cells at minimum dose, in order to control the toxicity towards normal cells^8^. Gamma rays, X-rays, and charged particles are the utmost types of radiation delivered by a machine outside the body, or irradiated through radioactive material placed near to the cancer cells/tissue in the body for cancer treatment. However, ionizing radiations are also used in agriculture, pharmaceutical, and other technological processes, rather than treatment of biological systems. Among all other ionizing radiations, Gamma ray use is more economical and effective, because of its easy availability and penetration power^9^. Moreover, the change in structural, morphological, and biological systems depends on the strength and duration of exposure of gamma doses. Over the last few years, non-thermal atmospheric pressure (NTP) plasma has been studied as the alternative to ionizing radiations for cancer treatments.

Plasma medicine is a new field that uses NTP plasma for a diversity of medical applications^10–12^, such as sterilization^12^, wound healing^13^, blood coagulation^14, 15^, and cancer treatment^16^. NTP plasma interacts with the oxygen, nitrogen, water, etc. in air, to produce various radical and non-radical species, for example, hydroxyl radicals (^•^OH), superoxide (O_2_^•−^), singlet oxygen (^1^O_2_), nitrogen dioxide (NO_2_), hypochlorite (ClO^−^), atomic oxygen (O), and nitric oxide (NO). During the plasma–liquid interactions, some relatively long lifetime reactive species are generated in liquid, such as hydrogen peroxide (H_2_O_2_), nitrites (NO_2_^−^), and nitrates (NO_3_^−^)^17, 18^. There are mainly two types of NTP plasma devices, i.e. plasma jet and dielectric barrier discharge, which display significant anti-cancer capacity in several cancer cell lines during *in vitro* studies^19–23^, and with several xenograft tumors for *in vivo* studies^24–27^.

Some of the cancer cell lines that are studied using NTP plasma, such as skin cancer^28^, breast cancer^29^, colorectal cancer^30^, lung cancer^31^, cervical cancer^32^, leukemia^33^, hepatoma^34^, as well as head & neck cancer^35^, reveal the selective anti-cancer treatment modality of NTP plasma treatment, in contrast to conventional anti-cancer approaches (chemotherapy, radiotherapy, and surgical excision). Similar to radiation treatments, NTP plasma treatments also damage the cells via an increase in the local concentration of reactive oxygen and nitrogen species (RONS). The increase in reactive oxygen species (ROS) also leads to induction of antioxidant defense mechanisms in the cell, such as glutathione peroxidase superoxide dismutase, catalase, superoxide dismutase, glutathione reductase, and glutathione peroxidase, as well as other small molecules, such as like NADPH and GSH and NADPH^36, 37^. Both the enzymatic defense in the cell cytosol compartment of CuZn-superoxide dismutase (CuZnSOD), and its mitochondrial counterpart of MnSOD^37^, were activated. A recent study shows that both the decreased and enhanced expression of Cu, Zn-SOD, or Mn-SOD influence the plasma-induced HeLa cell death^38^. However, the role of osmolytes in cancer treatment during plasma and gamma irradiation is still a mystery.

Most of the proteins adopt a tightly folded structure, signifying the lowest possible free energy of the polypeptide chain and the surrounding solvent under physiological conditions^39^. It has been reported that osmolytes are engaged in cell volume regulation in mammalian cells^40^. Some cells use organic osmolytes solutes, in addition to inorganic solutes, because high concentrations of inorganic salts (Na^+^ or K^+^) disturb the proteins function; however, organic solutes do not perturb the proteins function^41^. Under physiological conditions, proper protein conformation plays an important role in cell viability^41^. Different protein conformations depend upon the cellular and functional needs of the body^42^. In cells, the organic osmolytes accomplished the protein folding by shifting folding equilibrium, and they can manipulate the structure and conformation of proteins that provides the potential to ameliorate challenging diseases that occur due to misfolding of proteins. Protein misfolding challenges cell homeostasis through protein degradation or protein aggregation. In both cases, deleterious genetic disorders can happen. Therefore, osmolytes can stabilize the protein folding, and protein functions, and also shift the folding equilibrium away from the aggregation and/or degradation of proteins^43–46^. Therefore, to understand the action of gamma rays and NTP plasma on the proteins in the presence of organic osmolytes, we have used myoglobin as the model protein.

Myoglobin (Mr 16,700) is an oxygen binding protein that is found within muscle cells. Myoglobin protein crystal structure and its unfolding have been well studied^47^. The importance of myoglobin has been extensively discussed in most biochemistry texts^48^. Myoglobin contains a heme prosthetic group, and one polypeptide chain of 153 residues. The heme group resides deep inside the hydrophobic pocket within a protein’s structure. Decrease in the sorbet band has been observed due to denaturing effect of polar aqueous solvents on the heme which in turn results in the denatured myoglobin^49^.

Therefore, in this work we have treated the myoglobin protein with gamma rays and DBD NTP plasma in ambient air with or without osmolytes (sorbitol (500 mM) and trehalose (500 mM)), and checked the structural and thermodynamic properties, using CD spectroscopy and UV-vis spectroscopy. In addition, we have studied the reactive oxygen and nitrogen species (RONS) generated in phosphate buffer after the irradiation of gamma rays and DBD plasma. Further, we have studied the molecular dynamic simulation of myoglobin and hydrogen peroxide (H_2_O_2_) interactions with and without the osmolytes. This study provides information on the role of osmolytes in protein folding during gamma ray and DBD plasma treatments.

## Results and Discussion

Protein denaturation studies provide remarkable insight into the kinetics and thermodynamics of protein folding^50–59^. Chemical or temperature induced unfolding provides insight into the thermodynamic stability of a protein. Therefore, in the present study we study the chemical and thermal denaturation of Myoglobin proteins after Gamma ray and DBD plasma treatments.

### Change in absorbance of the protein after treatment with Gamma rays and DBD plasma in the presence of urea

The interaction of heme with histidine residue in the restricted pocket of fully-folded Myoglobin protein leads to an intense Soret band peak^49^. To illustrate myoglobin unfolding with increasing urea concentration, we studied the absorbance intensity at 409 nm. The high absorbance intensity is proportional to the concentration of folded protein molecules, and changes of absorbance at 409 nm correlate to the myoglobin unfolding. The absorbance at 409 nm in the spectra for native myoglobin in phosphate buffer is assumed to correspond to 100% folded myoglobin protein (A_f_) at 0 M urea concentration, and the absorbance at 409 nm with 9.0 M urea is assumed to the unfolded protein (A_u_).

$${\rm{Fraction}}\,{\rm{Folded}}=\frac{{A}_{409}-{A}_{u}}{{A}_{f}-{A}_{u}}$$

Here, the A_409_ is the absorbance at all concentrations of urea (except 0 and 9 M), and this was used to calculate the fraction folded. Figure S1 shows that without treatment with the Gamma rays, 50% fraction of proteins was denatured at 6.5 M urea. But after treatment with gamma rays at the absorbed dose of 228, 424, and 1,136 Gy, the 50% fraction of proteins was denatured at 6, 5.6, and 5 M of urea concentration, respectively. These results reveal that as the gamma rays absorbed dose increases, the myoglobin is denatured at lower urea concentration. The least denaturation occurred at the absorbed dose of 228 Gy, and the maximum denaturation occurred at the absorbed dose of 1,136 Gy as the function of urea concentration. When we added the 500 mM of sorbitol or trehalose to the Myoglobin protein solution, the 50% fraction of protein denaturation occurred at 6.5 M of urea concentration, for both osmolytes. Hence, the fraction of protein denatured in the presence of urea is the same for Myoglobin protein with or without osmolytes. So later, we have treated the protein with gamma rays at different intensity, in the presence of sorbitol and trehalose, respectively. Figure S1 shows that the 50% fraction of protein was denatured at 5.2, 5.8, and 6.1 M of urea in the presence of sorbitol, and 5.6, 6, and 6.3 M of urea in the presence of trehalose, for the absorbed dose of 1,136, 424, and 228 Gy, respectively.

Further, we have treated the Myoglobin protein with DBD plasma in the ambient air, containing 40% humidity. We observed that after treatment of 5, 10, and 20 min of DBD plasma, the 50% fraction of protein was denatured at 6, 5.6, and 4.5 M of urea, as shown in Figure S2. Whereas for the protein solution in the presence of sorbitol (500 mM), when treated with DBD plasma for 5, 10, and 20 min, the 50% fraction of protein was denatured at 6, 5.8, and 5.5 M of urea. On the other hand, in the presence of 500 mM trehalose, the 50% fraction of protein was denatured at 6.2, 6.0, and 5.6 M of urea, after treatment of DBD plasma for 5, 10, and 20 min (Figure S2). This shows that in both treatments, of gamma rays and DBD plasma, the structure of Myoglobin denatured at lower concentration of urea, as the absorbed dose of gamma rays increases, or the treatment time of DBD plasma increases. But in the presence of osmolytes, the structure of protein was denatured at higher concentration of urea as compared to without osmolytes after the treatment with gamma rays and DBD plasma, respectively.

### Thermal denaturation of Myoglobin protein after the treatment with gamma rays and DBD plasma

Well-defined three-dimensional structures of proteins commonly occur only within the bounds of specific environmental conditions. Outside the environmental boundaries, proteins display an unfolded state. Many research groups used CD spectroscopy to understand the melting temperature or transition temperature (T_m_) of proteins/enzymes^56, 57, 60, 61^. Therefore, in our study we have used CD spectroscopy to understand the change in T_m_ of protein in the presence of osmolytes; and also, the impact of gamma rays and DBD plasma on the change of T_m_ of protein, with or without osmolytes.

**Figure 1:**
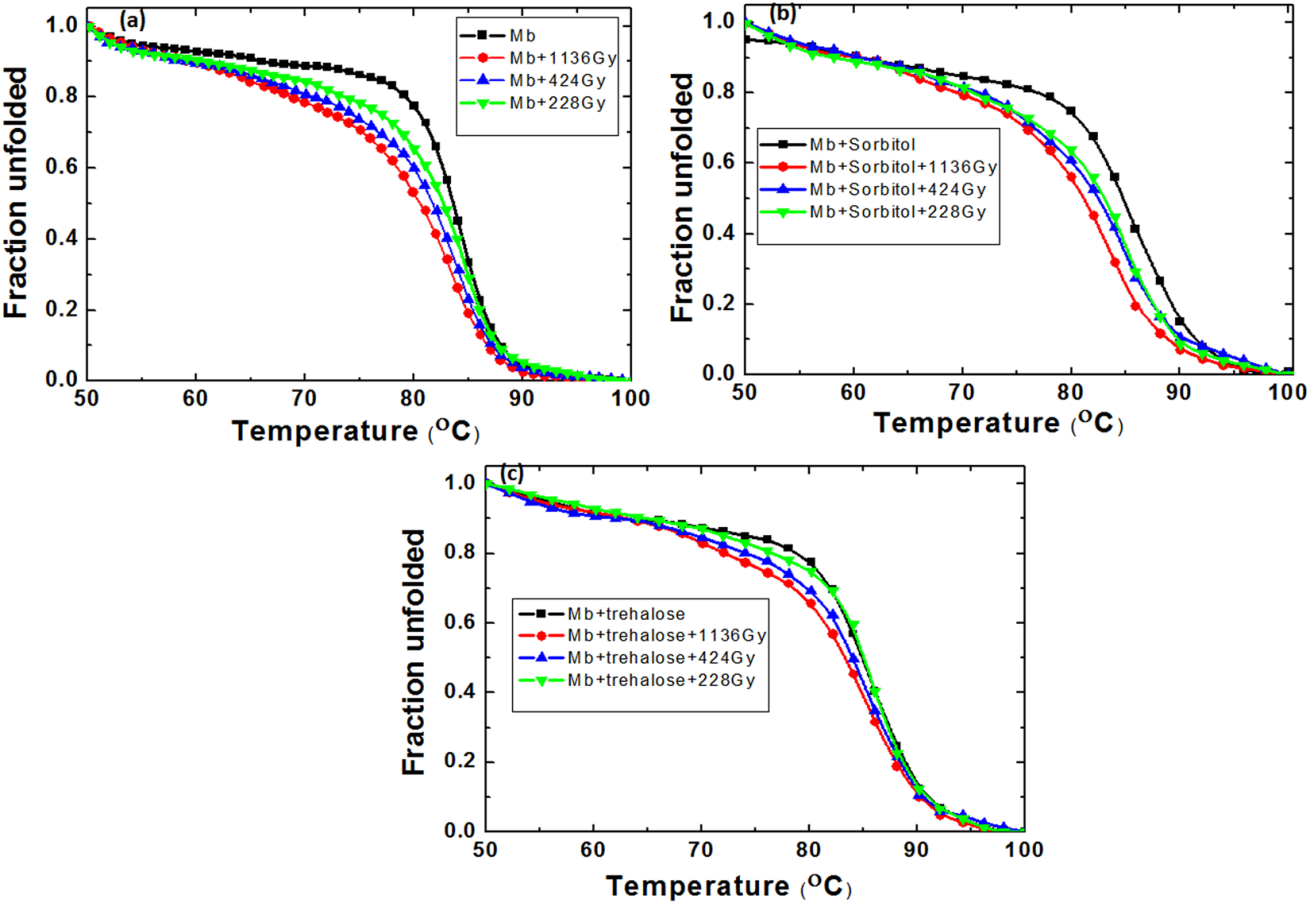
Thermal denaturation profile of Myoglobin protein after treatment with different gamma rays absorbed dose such as 1136, 424 and 228 Gy (**a**) control Myoglobin, (**b**) Myoglobin + sorbitol and (**c**) +trehalose.

Firstly, we have treated the myoglobin with gamma rays, and we observed at absorbed dose of 1,136, 424, and 228 Gy, the T_m_ of protein was 80, 81, and 82 °C, while the T_m_ of control protein (without gamma treatment) was 83 °C (Fig. 1a). On the other hand, when we have added the sorbitol (500 mM) to the myoglobin protein, the T_m_ was increased to 85 °C. Figure 1b shows that after the gamma irradiation on the solution containing the sorbitol and myoglobin, the T_m_ values were 81, 82.5, and 83 °C, at 1,136, 424, and 228 Gy, respectively. Nevertheless the addition of trehalose to the myoglobin sample solution increased the T_m_ value to 85.5 °C. Figure 1c shows that the changes in the T_m_ values of trehalose containing solution after the gamma rays treatment were 83, 83.5, and 84 °C at 1,136, 424, and 228 Gy absorbed dose, respectively. These results show that both osmolytes, i.e. sorbitol and trehalose, can protect the protein structure, even in the presence of gamma rays. But between the osmolytes, the trehalose has more counteraction tendency to control the myoglobin structure, even at high gamma absorbance dose.

**Figure 2:**
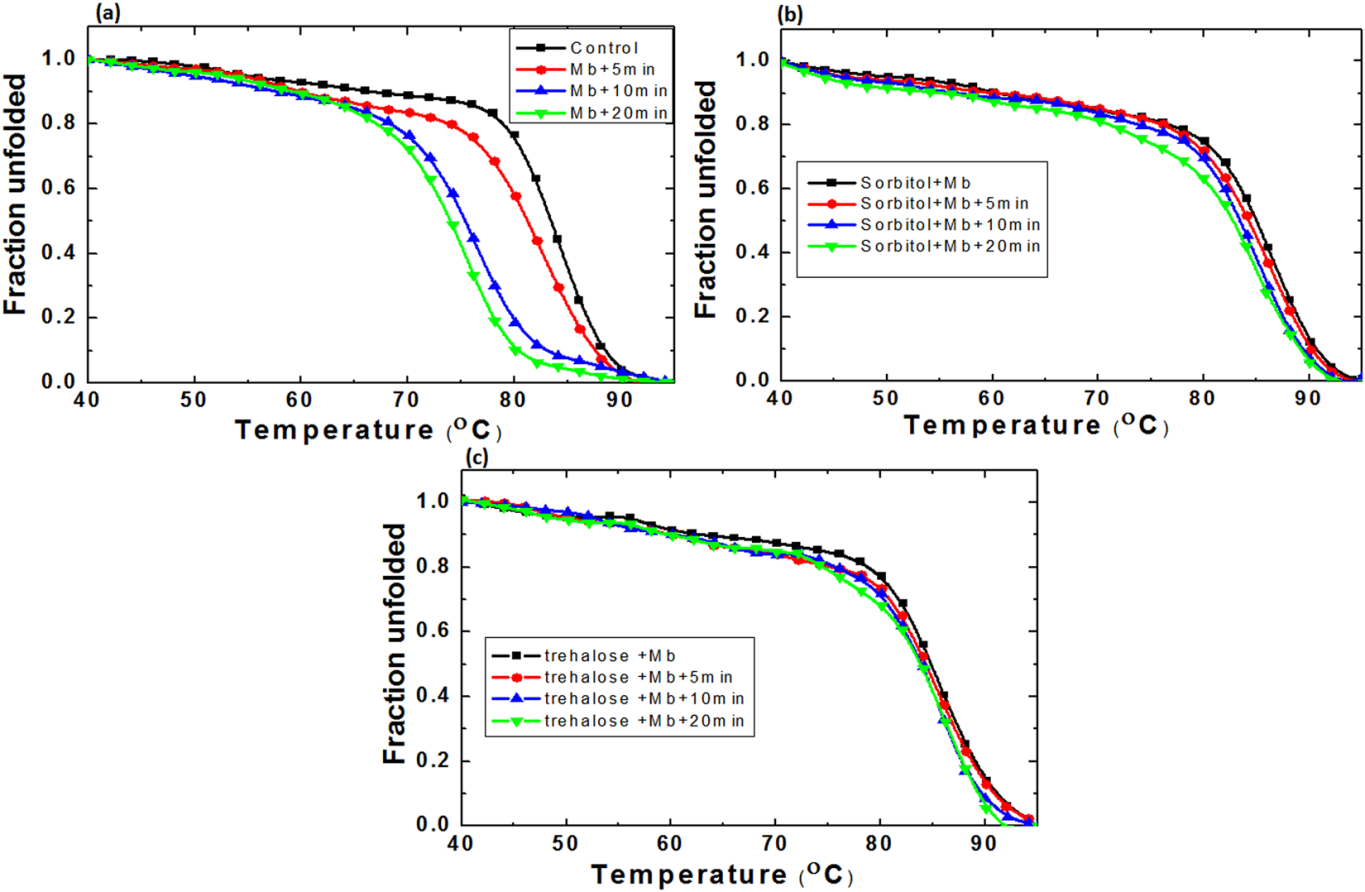
Thermal denaturation profile of Myoglobin protein after treatment with DBD plasma for different time intervals such as 20, 10 and 5 min (**a**) control Myoglobin, (**b**) Myoglobin + sorbitol and (**c**) +trehalose.

Later, to understand the effect of osmolytes on the T_m_ values of proteins in the presence of DBD plasma, we have studied the thermal denaturation. We observed that the myoglobin thermodynamics has changed after the treatment with DBD plasma for 5, 10, and 20 min. Figure 2a shows that after treatment with DBD for 5, 10, and 20 min, the T_m_ value of protein changes to 81, 75, and 74 °C, respectively. Further, we observed that after 5, 10, and 20 min treatment of DBD plasma in the presence of sorbitol, the T_m_ value of protein changes to 84.5, 83.6, and 83 °C, respectively (Fig. 2b). However, Fig. 2c shows that the addition of trehalose drastically changes the T_m_ values after treatment of DBD plasma for 5, 10, and 20 min to 84, 83.8, and 83.7 °C, respectively. These results show only a ≈1 °C decrease in the T_m_ value of protein, even after 20 min DBD plasma treatment in the presence of trehalose.

If we compare the change in T_m_ of control myoglobin protein (without osmolytes) after gamma rays and DBD plasma irradiations, we observe that in the case of DBD treatment, the changes in T_m_ values are greater, as compared to the case of gamma ray treatments. However, in the presence of osmolytes (sorbitol and trehalose), the T_m_ value of protein has increased from 83 to 85 °C. When the gamma rays are irradiated on the samples containing osmolytes, the decrease in T_m_ values is much less (about ≈4 °C for sorbitol and ≈2 °C for trehalose for the highest studied absorbed dose of 1,136 Gy). Meanwhile, the decrease in T_m_ values after the DBD plasma treatment for 20 min in the presence of sorbitol is ≈2 °C, and for trehalose is ≈1 °C. These results reveal that osmolytes can control the denaturation action of protein after the DBD treatment better than after the gamma treatments. The possible reason for this process will be discussed in our latter sections.

### Structural changes of myoglobin protein after gamma and DBD plasma treatments with or without urea

**Figure 3:**
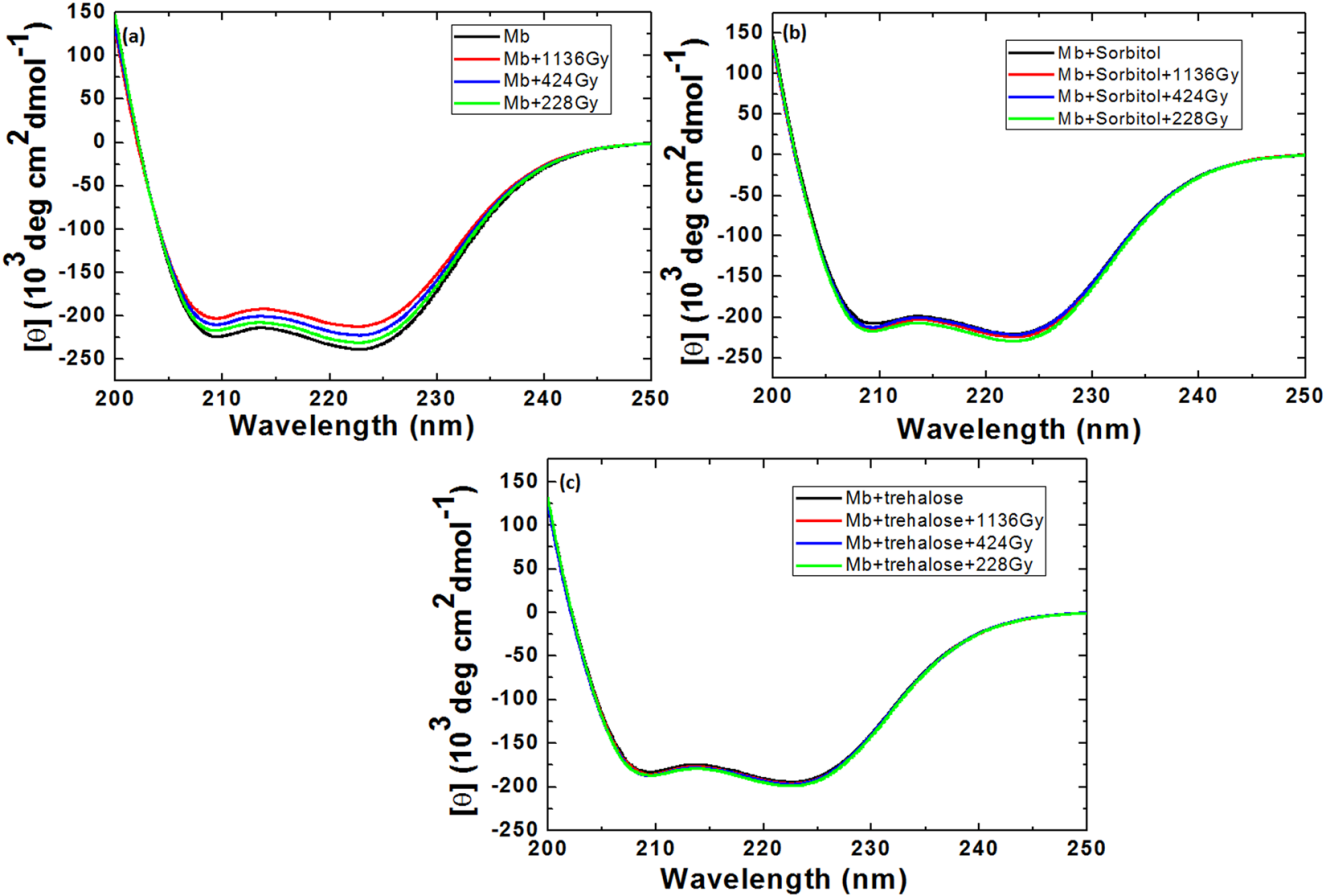
Analysis of Myoglobin secondary structure change using CD spectroscopy after treatment with different gamma rays absorbed dose such as 1136, 424 and 228 Gy (**a**) control Myoglobin, (**b**) Myoglobin + sorbitol and (**c**) +trehalose.

**Figure 4:**
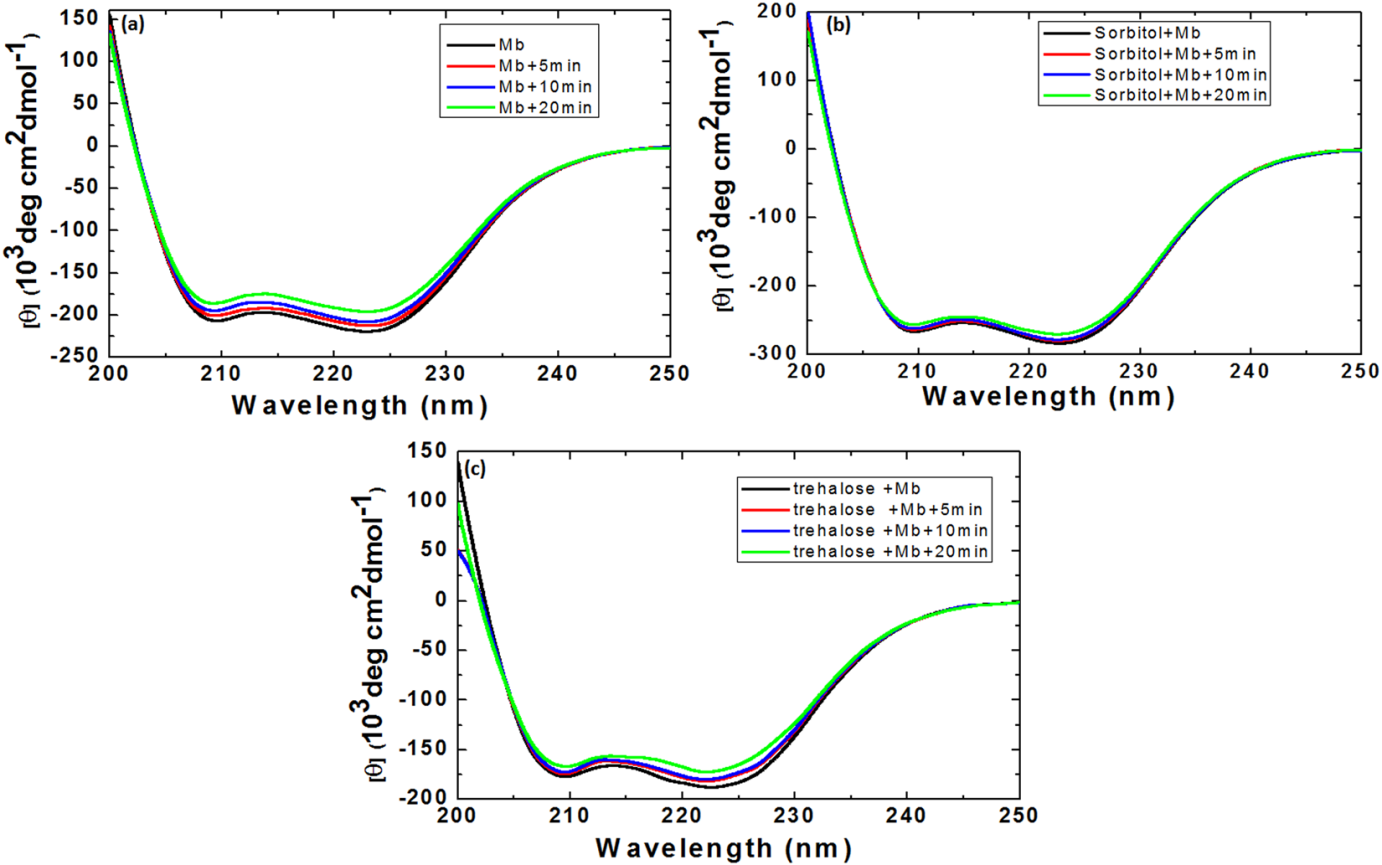
Analysis of Myoglobin secondary structure change using CD spectroscopy after treatment with DBD plasma for different time intervals such as 20, 10 and 5 min (**a**) control Myoglobin, (**b**) Myoglobin + sorbitol and (**c**) +trehalose.

To understand the structural modification in myoglobin protein after treatment with gamma rays and DBD plasma, we further executed CD experiments. Figures 3 and 4 show that the far-UV CD spectrum of myoglobin shows distortion in the secondary structure of protein after different treatments. The displayed CD spectra of myoglobin have two well-pronounced minima at ≈210 and ≈222 nm that resemble those typical for the polypeptide chain that is mostly organized in α-helix conformation. Figure 3, reveals that after the treatment with the gamma rays at absorbed dose of 1,136, 424, and 228 Gy the α-helical structure of myoglobin was 54%, 57%, and 58%, respectively; whereas, the control myoglobin structure has α-helical of 63%. After the addition of sorbitol (500 mM) to the myoglobin solution, the α-helical is increased to 64%. Later, Fig. 3 shows that the percentage of α-helical changes to 55%, 56%, and 58%, after the treatment with gamma rays at the absorbed dose of 1,136, 424, and 228 Gy, respectively. Moreover, the change of α-helical structure in myoglobin with the addition of 500 mM trehalose is 65%. Further, the treatment of myoglobin protein solution in the presence of trehalose with gamma rays at the absorbed dose of 1,136, 424, and 228 Gy shows the change in α-helical structure to 58%, 59%, and 60%, respectively.

Figure 4 shows that, we have treated the myoglobin protein structure with the DBD plasma for 5, 10, and 20 min, and observed the change in α-helical structure of protein. After the treatment of myoglobin protein for 5, 10, and 20 mins, the % of α-helical changes to 60%, 59% and 57%, respectively. Latter, the addition of osmolytes increases the α-helical structure of protein, as seen above. To understand the impact of DBD plasma irradiation on the secondary structure of protein in presence of osmolytes, we observed the change in α-helical structure. The α-helical structure changes to 63%, 60% and 57%, in the presence of sorbitol + Myoglobin + 5, +10 and +20 min DBD plasma treatments, respectively. On the other hand, the addition of trehalose with DBD plasma treatment changes the α-helical structure to 62%, 61% and 59%, after treatment for 5, 10 and 20 min, respectively.

Figures S3 and S4 shows the impact of the various urea concentrations on the α-helical structure of protein at different conditions. We observed the peak at ≈222 nm as the function of urea concentration. So, as the urea concentration increases, the ellipticity value decreases (positive ellipticity). The ellipticity value of myoglobin decrease (positive ellipticity) slightly up to 4 M urea; later, between 5 and 8 M urea, the slope is quite steep; but then becomes constant at 9 M urea (Figure S3). At the absorbed dose of 1,136, 424, and 228 Gy, the ellipticity values are higher than those of the myoglobin protein control sample for 0 to 8 M concentration; at 9 M urea, all of the values meet each other. For Myoglobin + sorbitol and +trehalose, the ellipticity values decrease (positive ellipticity) slightly up to the 5 M urea; but between 5 and 8 M urea, the slope is quite steep; but then becomes constant at 9 M urea. In both cases (Myoglobin + sorbitol and +trehalose), the gamma-treated samples in the presence of sorbitol or trehalose are higher than their corresponding control values. As the absorbed dose (1,136, 424 and 228 Gy) increases, the ellipticity also decreases (positive ellipticity) at all concentrations of urea except for 9 M; this might be because at 9 M urea, the structure of myoglobin denatures completely (as previously measured), so no change in ellipticity observed. The difference of ellipticity among different gamma absorbed dose is least for the trehalose solution, slightly higher for the sorbitol solution, and quite high for the control solution (without osmolytes).

Figure S4 shows that we have checked the impact of the various urea concentrations on the α-helical structure of myoglobin protein in the presence of DBD plasma treatments. We observed a peak at ≈222 nm as a function of urea concentration. So, as the concentration of urea increases, the CD ellipticity values for myoglobin decrease (positive ellipticity), similar to the gamma ray treatments. The ellipticity values decrease (positive ellipticity) slightly up to 5 M urea, but the slopes are quite steep between 6 and 8 M urea; and then become constant at 9 M urea, for DBD plasma treatments at 5, 10, and 20 min (Figure S4). In the presence of osmolytes + protein (Myoglobin + sorbitol and Myoglobin + trehalose) for the DBD plasma-treated samples, the ellipticities at 222 nm are higher than their corresponding control values. As the DBD treatment time increases, the CD ellipticity values for protein decreases (positive ellipticity) at all concentrations of urea, except for 9 M. The difference of ellipticity among different treatment conditions in the presence of different osmolytes is least for the trehalose solution. The impact of DBD plasma treatments as a function of urea concentration doesn’t change the ellipticity values much for the trehalose solutions, as compared to the other solutions. Hence, trehalose solution protects the protein secondary structure from DBD plasma treatments in the best way, as compared to the other conditions of treatment (sorbitol solution or control solutions). Therefore, to understand the reason for the structural change after gamma ray and DBD plasma treatments, we have studied the generation of reactive species in buffer solution.

### Analysis of reactive species generated and change in pH in solution after the treatment with gamma rays and DBD plasma

**Figure 5:**
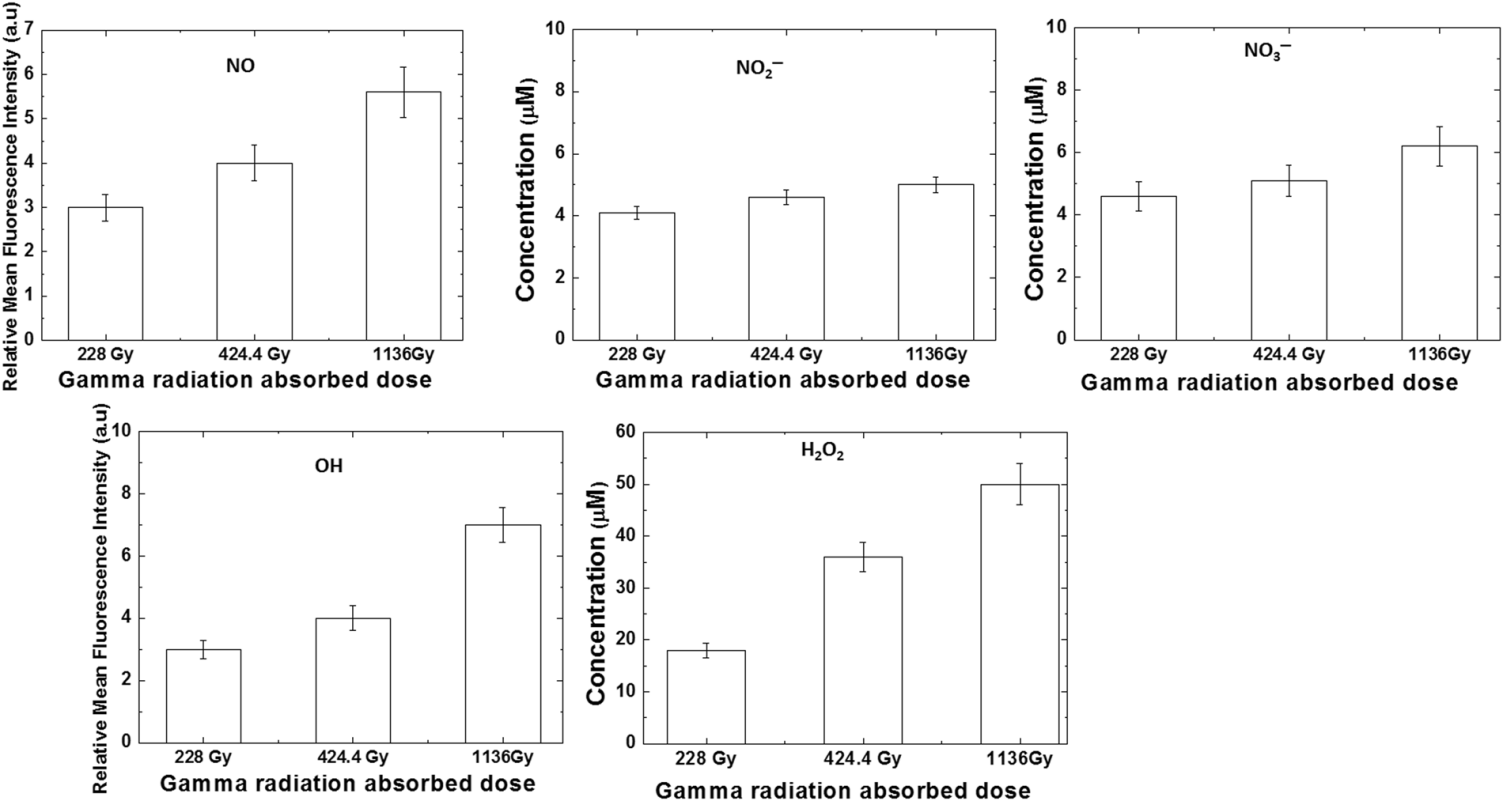
Analysis of reactive oxygen and nitrogen species detection in phosphate buffer after treatment with different gamma rays absorbed dose such as 1136, 424 and 228 Gy.

**Figure 6:**
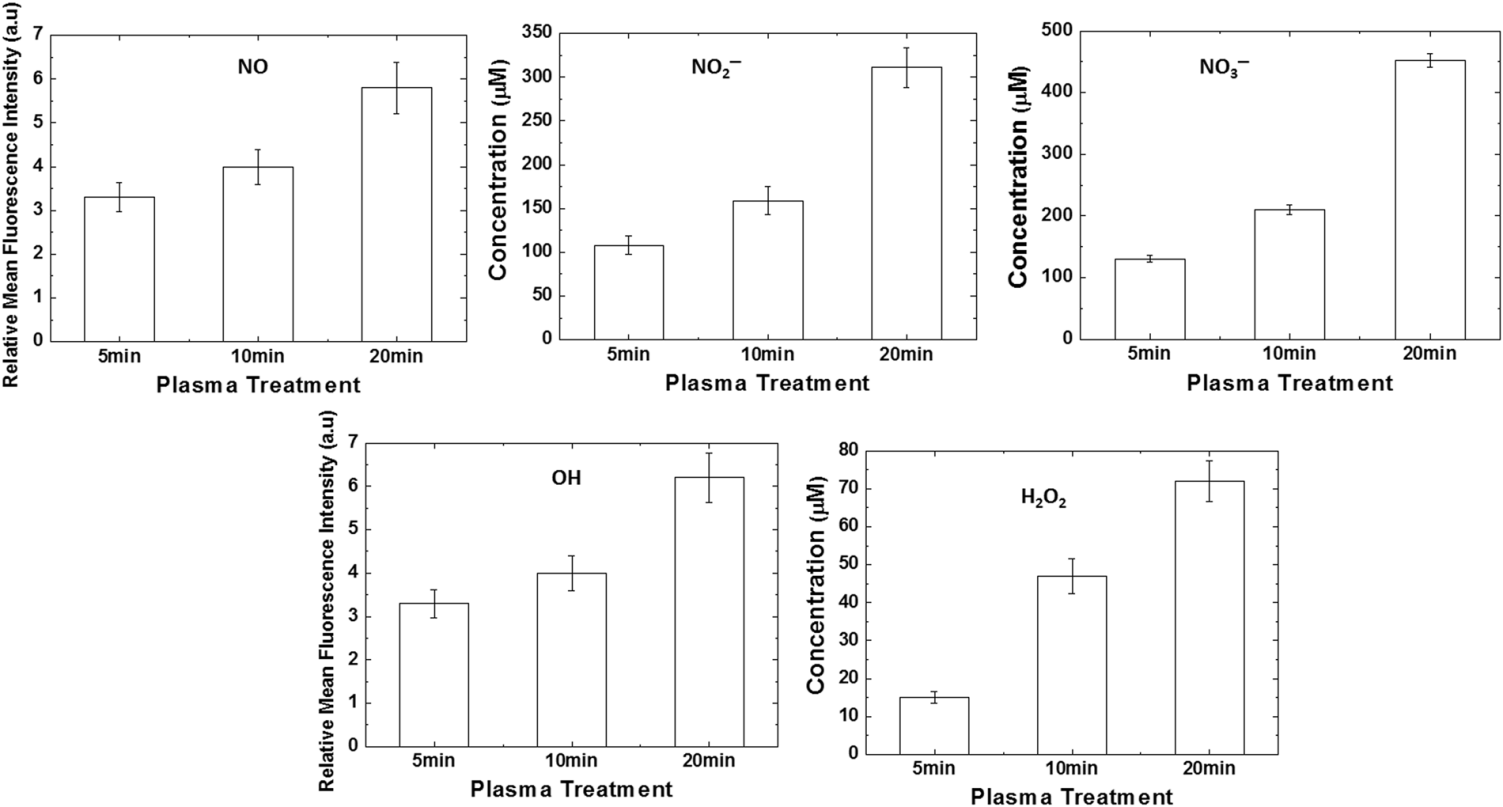
Analysis of reactive oxygen and nitrogen species detection in phosphate buffer after treatment with DBD plasma for different time intervals such as 20, 10 and 5 min.

The irradiation of gamma rays and DBD plasma on buffer results in the formation of reactive nitrogen and oxygen species (RNS and ROS). Figures 5 and 6 show the results of the chemical analysis that we have performed to determine the amount of reactive species generated in solution after different treatments (gamma rays and DBD plasma). During this chemical analysis, we have studied the formation of ROS, such as ^•^OH and H_2_O_2_, against the RNS, such as NO^•^, NO_2_^−^, and NO_3_^−^. After the gamma rays treatment on the buffer, we analysed the ^•^OH and NO^•^. We observed from Fig. 5 that as we increased the gamma radiation absorbed dose from 228, through 424, to 1,136 Gy, the fluorescence intensity also increases for both ^•^OH and NO^•^ radicals. This shows that a greater absorbed dose results in the ability to generate more radicals in buffer solution. Figure 6 show that we have later analyzed the change in fluorescence intensity for both ^•^OH and NO^•^ radicals after DBD plasma treatments for 5, 10, and 20 min. The figure shows similar trends to those of the gamma ray treatments: as the treatment time increases, the fluorescence intensity also increases for both radicals. Hence, the intensity of radicals is directly related to the treatment time; more treatment time results in more generation of ^•^OH and NO^•^ radicals in buffer.

Additionally, we have analyzed the concentration of H_2_O_2_, NO_2_^−^, and NO_3_^−^ after the gamma irradiation in buffer for the absorbed dose from 228, 424, and 1,136 Gy. We observed that the H_2_O_2_ concentration for 228, 424 and 1,136 Gy absorbed dose was ≈18, 36, and 50 μM, respectively. Whereas, Fig. 5 shows that the NO_2_^−^ concentration was ≈4.1, 4.6, and 5 μM, and the NO_3_^−^ concentration was ≈4.6, 5.1, and 6.2 μM for the absorbed dose of 228, 424, and 1,136 Gy, respectively. Similar to the behavior of ^•^OH and NO^•^ radicals in buffer, the H_2_O_2_, NO_2_^−^, and NO_3_^−^ concentration also increases as the gamma radiation absorbed dose increases. However, after the same gamma radiation absorbed dose treatment, the concentrations of NO_2_^−^ and NO_3_^−^ were quite low, as compared to the H_2_O_2_ concentration. This might be because of water radiolysis by γ-rays that generate OH radicals^62^, which react with other OH radicals to produce the H_2_O_2_. On the other hand for the NO^•^ radicals, NO_2_^−^, and NO_3_^−^ are generated, because of the presence of air molecules during the gamma ray irradiation.

Moreover, we have also analyzed the concentration of H_2_O_2_, NO_2_^−^, and NO_3_^−^ after the DBD plasma treatment on buffer for 5, 10, and 20 min. We observed that the H_2_O_2_ concentration for 5, 10, and 20 min DBD plasma treatment was ≈15, 47, and 72 μM, respectively. Whereas, Fig. 6 shows that the NO_2_^−^ concentration was ≈108, 159, and 311 μM, and the NO_3_^−^ concentration was ≈130, 210, and 452 μM for 5, 10, and 20 min DBD treatments, respectively. Similar to the gamma ray treatment, the behavior of H_2_O_2_, NO_2_^−^, and NO_3_^−^ concentration increases as the DBD plasma treatment time increases. However at each DBD plasma treatment time, the concentrations of NO_2_^−^ and NO_3_^−^ were quite high, as compared to the H_2_O_2_ concentration. The generated OH radicals are due to plasma interaction with water molecules in air and on the surface of buffer; when these OH radicals react with each other, they produce H_2_O_2_ (similar to the gamma treatments). On the other hand, the NO^•^ radicals, NO_2_^−^ and NO_3_^−^ ions are generated due to the presence of air. If we compare the H_2_O_2_ production in buffer after gamma ray irradiation and plasma irradiation, we observe that in plasma there is slightly more H_2_O_2_ produced, than in gamma ray treatment. While more NO_2_^−^ and NO_3_^−^ ions are generated in plasma than in gamma ray, this might be due to the different environmental conditions for the two treatments. We cannot say anything about the environmental condition in the gamma rays treatment chamber (as we are unable to measure the environmental condition during treatment), while the DBD plasma treatments were performed at atmospheric conditions (air as feeding gas, with humidity of 40%). The difference in environmental condition during treatments might be the reason for the high RNS species generated for DBD plasma, but the amounts of H_2_O_2_ produced for gamma rays and DBD plasma are approximately similar to each other. Hence, H_2_O_2_ generation during gamma ray and DBD plasma treatment can be the main component for the structural changes of myoglobin protein. Additionally, we have not observed any change in pH and temperature of the solution after treatment from gamma rays or DBD plasma (data not shown). Further, we have studied the molecular dynamic simulation of myoglobin protein with H_2_O_2_ in the presence of sorbitol and trehalose, respectively for better understanding the interaction properties.

### Molecular dynamics simulation of myoglobin in the presence of H_2_O_2_ with or without osmolytes

**Figure 7:**
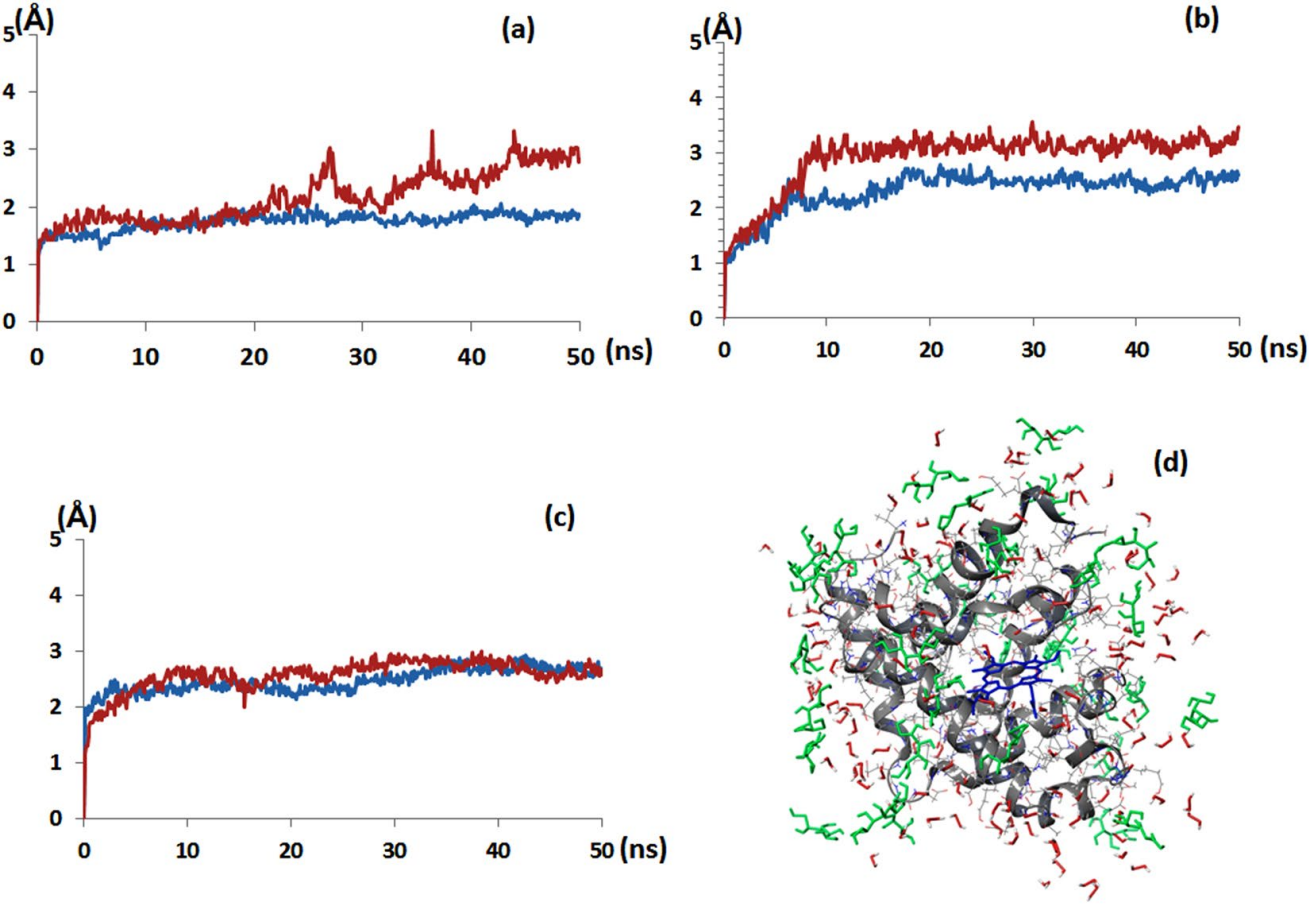
Molecular dynamics simulation result of Myoglobin, (**a**) RMSD plots of the Myoglobin in the phosphate buffer and 21% H_2_O_2_; (**b**) RMSD plots of the Myoglobin in the phosphate buffer, 21% H_2_O_2_ + 21% sorbitol; (**c**) RMSD plots of the Myoglobin in the phosphate buffer, 21% H_2_O_2_ + 21% trehalose; (**d**) Snapshot of Myoglobin with 21% sorbitol + 21% H_2_O_2_ (Gray ribbon and wire - myoglobin, Blue - heme group, Green - sorbitol and Red - H_2_O_2_).

To understand the influence of osmolytes on the myoglobin protein, and how the presence of osmolytes can affect the denaturation action of hydrogen peroxide (H_2_O_2_) on myoglobin protein structure, we analyzed the structural change of these paralogs using MD simulations. We prepared three types of MD simulation systems: with the protein solvated in phosphate buffer (21 mM) and 21% H_2_O_2_, with or without osmolytes (sorbitol and trehalose)_._ Through measuring values of the root-mean-square atomic positional deviation (RMSD), we studied the structural stabilities of myoglobin for each environmental condition. Although, H_2_O_2_ molecules were uniformly distributed in solutions, but they seemed to condense within 1 Å of the surface of protein in a very short duration. Figure 7 shows that we subsequently measured the RMSD values at all conditions. However, in the presence of 21% H_2_O_2_, myoglobin underwent conformational changes, and showed higher RMSD values than for the simulation without H_2_O_2_ after the simulation for 50 ns. The RMSD value controlling myoglobin protein was 1.76, but after the treatment with H_2_O_2_, it was increased to 2.18, with change in standard deviation value of 0.29, as seen in Table 1. So, we may conclude that the presence of H_2_O_2_ destabilizes the myoglobin structure on the basis of these results. Later, we studied the MD simulation to understand the action of the osmolytes sorbitol and trehalose on myoglobin, with or without H_2_O_2_. Figure 7 show that Myoglobin in the presence of 21% sorbitol or 21% trehalose with phosphate buffer solution maintained stable conformations for 50 ns. Additionally, we have also measured the RMSD for sorbitol-buffer solution with H_2_O_2_. The RMSD value for controlling myoglobin protein with sorbitol was 2.31, but after the treatment with H_2_O_2_, it was increased to 2.92, with change in standard deviation value of 0.15 (Table 1). Later, we have checked the RMSD for trehalose-buffer solution with H_2_O_2_. We observed that the RMSD value for controlling myoglobin protein with trehalose was 2.47, but after the treatment with H_2_O_2_, it was increased to 2.57 with change in standard deviation value of 0.07, as seen in Table 1. Therefore, we conclude that in the presence of H_2_O_2_, trehalose can stabilize the protein more than sorbitol can.

**Table 1:** Root-mean-square atomic positional deviation (RMSD) and standard deviation of Myoglobin, Myoglobin + sorbitol and +trehalose.

	Myoglobin	Myoglobin + sorbitol	Myoglobin + trehalose
**RMSD**			
** Without H**_**2**_ **O**_**2**_	1.76	2.31	2.47
** With H**_**2**_ **O**_**2**_	2.18	2.92	2.57
**stdev**			
** Without H**_**2**_ **O**_**2**_	0.17	0.37	0.23
** With H**_**2**_ **O**_**2**_	0.46	0.52	0.30
** Δ**	0.29	0.15	0.07

The protective action of sorbitol and trehalose against the gamma rays and DBD plasma treatments can be explained as the scavenging action of these osmolytes against RONS. In the previously reported articles, it was observed that sorbitol has scavenging activities for ^•^OH inhibition^63, 64^. And in another study authors reported that sorbitol can prevent the DNA cleavage against the ^•^OH attach, through its ^•^OH-scavenging property^65^. Additionally, it was observed that trehalose can protect the DNA against the γ and β irradiations^66^. Another study reveals that trehalose assists as the free radical scavenger, they also observed that trehalose can protect HepG2 cells against the palmitate-induced toxicity^67^. Although it was also reported that trehalose can protect the cells against the oxygen radicals. And also trehalose can enhance the resistance to cells towards oxidative damage caused by H_2_O_2_^68^. Hence, these all studies support that both osmolytes (sorbitol and trehalose) have the tendency to protect the biological systems against the RONS action.

## Conclusion

In our present study, we have checked the thermodynamics and secondary structure of myoglobin protein with and without osmolytes after the gamma ray and DBD plasma treatment. The thermodynamics of myoglobin protein has been studied as a function of urea concentration, as well as a function of temperature. We observed that the denaturation of protein as a function of urea concentration synchronized with the treatments (gamma rays and plasma). After treatment with gamma rays and plasma, the myoglobin denatures at lower urea concentration. Similarly, the denaturation of myoglobin as a function of temperature also varies after the treatment with gamma rays and DBD plasma. The T_m_ values of protein decrease more after both treatments without osmolytes. However, the addition of osmolytes, such as sorbitol and trehalose, control the structure of myoglobin, even after the irradiation of gamma and plasma. Among both osmolytes, the trehalose protects the structure of myoglobin against both treatments (gamma and plasma) better than does sorbitol. However, the H_2_O_2_ concentrations generated in buffer after gamma and plasma treatments do not have much difference, but the NO_2_^−^ and NO_3_^−^ concentration values are quite higher for the plasma treatment than for the gamma treatments. But the denaturation still occurs slightly higher for gamma ray treatment, as compared to plasma treatment, even in the presence of osmolytes. Therefore, we conclude that the gamma ray action of treatment is different from that of plasma, in that gamma rays can penetrate the protective action of osmolytes. But still both osmolytes can protect the protein conformation against the gamma and plasma treatments, and trehalose shows higher ability to protect than sorbitol. Moreover, MD simulation results also show that trehalose can better counter the denaturation action of H_2_O_2_, than can sorbitol. These findings can play an important role in plasma medicine to understand the treatment mechanism.

## Experimental section

### Materials

The Myoglobin protein, phosphate buffer (pH = 7.2), sorbitol, and trehalose were supplied by Aldrich Chemical Co. (USA). All chemicals and reagents were used without further purification. The concentrations of OH, NO, H_2_O_2_, NO_2_^−^, and NO_3_^−^ were measured by methods provided in our previous work^69–72^. The NO is detected using 4-amino-5-methylamino-2′,7′-difluorofluorescein (DAF-FM), the excitation/emission maxima is 495/515 nm for the DAF-FM-T (product of the reaction between the NO and DAF-FM). The detection of ^•^OH can be done using terephthalate anions obtained by mixing terephthalic acid (TA) in alkaline aqueous solution. These terephthalate anions react with ^•^OH to create highly fluorescent hydroxyterephthalate ions (HTA) and excitation/emission maxima are 310/425 nm for the formation of HTA. The H_2_O_2_ concentration is measured using titanyl ions in the presence of sodium azide to control the H_2_O_2_ degradation by nitrites. The NO_2_^−^ concentration is measured using Griess reagent supplied by Aldrich Chemical Co. (USA), whereas the NO_3_^−^ concentration is obtained using the Acorn Series ION 6 meter (pH/mV/°C Meter), nitrate electrode, from Oakton Instruments, USA.

### Dielectric barrier discharge

Experiments were carried out using a scalable DBD device, as described in previous research articles^73, 74^. The device was set in ambient air at atmospheric pressure. DBD plasmas were generated between the electrodes by supplying a 10 kHz AC high voltage (Logy Electric, LHV-09K). The discharge voltage and current were measured by high-voltage probe (Tektronix, P6015A) and a Rogowski coil (URD, CTL-28-S90–05Z-1R1), respectively. The peak-to-peak discharge voltage and current were 9.2 kV and 0.2 A. The corresponding discharge power density was 1.49 W/cm^2^, which was deduced from a voltage/charge Lissajous plot.

### Circular dichroism spectroscopy

CD spectroscopic studies were performed using J-815 spectrophotometry (Jasco, Japan) equipped with a Peltier system to control the temperature^52–56^. The samples were pre-equilibrated at the desired temperature for 15 min, and the scan speed was fixed for adaptive sampling (error F 0.01) with a response time of 1 s with 1 nm bandwidth. The secondary myoglobin structures were monitored using a 1.0 mm path length cuvette. The concentration for the secondary myoglobin structure was 0.2 mg/ml, with each spectrum being the average of six spectra. Each sample spectrum was obtained by subtracting the appropriate blank media without myoglobin from the experimental protein spectrum. The percentages of secondary structures were then calculated using Yang’s method^69^.

### Circular dichroism spectroscopy based urea studies

The stability studies were performed by temperature-controlled Jasco J-815 circular dichroism (CD) spectrometry. For each sample, the CD spectra were simultaneously measured from 200 to 250 nm at 25 °C. The ellipticity in the spectrum of native myoglobin in buffer was assumed to correspond to 100% folded protein, and the ellipticity in the spectrum of myoglobin with 9.0 M urea was assumed to correspond to unfolded protein. The displayed CD spectra of myoglobin have two well-pronounced minima at ≈210 and ≈222 nm that resemble those typical for the polypeptide chains that are mostly organized in α-helix conformation, so we have studied the change in ellipticity at 222 nm, to understand the change in α-helix conformation as the function of urea concentration.

### Temperature stability studies

Preliminary thermodynamic stability studies were performed by temperature-controlled J-815 spectrophotometry (Jasco, Japan) equipped with a Peltier system. For each sample, CD spectra were simultaneously measured at 222 nm as the function of temperature 25 to 100 °C. The sample was placed in a sealed cuvette to prevent water evaporation. The 222 nm ellipticity in the spectrum of native myoglobin in buffer at 25 °C was assumed to correspond to 100% folded protein, and at 100 °C was assumed to be unfolded protein, and the folded fraction was computed as:

$${\rm{Fraction}}\,{\rm{Folded}}=\frac{{A}_{222}-{A}_{u}}{{A}_{f}-{A}_{u}}$$

In this method, A_222_ is the absorbance between 25 and 100 °C, A_u_ is the absorbance of the unfolded protein at 100 °C, and A_f_ is the absorbance of the folded protein at 25 °C. We have studied the change in ellipticity at 222 nm, to understand the change in protein conformation as a function of temperature.

### UV-visible spectroscopy

UV-Vis S-3100 Spectrophotometry having a wavelength resolution of 0.95 nm, wavelength accuracy of ±0.5 nm, and wavelength reproducibility of ±0.02 nm was utilized for the analysis. A 0.5 mg/ml concentration of sample was used for UV-vis spectroscopy. The heme moiety in myoglobin exhibits a strong absorbance band at 409 nm that also displays a strong positive signal^49^. The 409 nm absorbance can be used to quantify the fraction of the folded protein. The fraction of folded protein was computed from the 409 nm absorbance following previous reports^59^. Briefly, the 409 nm absorbance in the spectrum of native myoglobin in buffer was assumed to correspond to 100% folded protein, and the 409 nm absorbance in the spectrum of myoglobin with 9.0 M urea was assumed to correspond to 0% folded protein (similar to the previous work ref. 75). The folded fraction was computed as:

$${\rm{Fraction}}\,{\rm{Folded}}=\frac{{A}_{409}-{A}_{u}}{{A}_{f}-{A}_{u}}$$

In this method, A_409_ is the absorbance, A_u_ is the 409 nm absorbance of the unfolded protein, and A_f_ is the absorbance of the folded protein.

### pH and temperature measurement

After the plasma was exposed for 5, 10, and 20 min in buffer, the pH and temperature of solution were measured using Horiba scientific instruments. All measurements were carried out in triplicate.

### Molecular dynamics simulation

Crystal structures of the human myoglobin were obtained from the (RCSB) protein data bank website (http://www.rcsb.org; the PDB IDs were 1MBN)^69^. These structures were treated with the Protein Preparation Wizard of the Schrödinger suite for molecular dynamics (MD) simulations. All water and heat molecules were eliminated, and hydrogens were added and minimized using IMPACT 6.6^69^. MD simulations were performed using Desmond 4.4^69^. Simulation systems were prepared with the Desmond system builder. The box shape of the systems was orthorhombic, and the size was determined by using the 10 Å buffer distance between the solute structures and the simulation box boundary. The systems were solvated with TIP3P model water. Na+ or Cl− ions were added to the systems to neutralize the total charges of the system. Hydrogen peroxide (H_2_O_2_) molecules were added to achieve 21% concentration of the systems. The charges of H_2_O_2_ were determined from the electrostatic potential (ESP) charge fitting with Jaguar 8.7^69^, version 8.7 (Schrödinger, LLC, New York, NY, 2015), using the basis set and function of B3LYP/6-31 G**. The MD simulations were performed in the (NPT) ensemble with the OPLS2005 all atom force field. A reference temperature of 300 K and pressure of 1 atm were maintained by the Nose-Hoover thermostat and the Martyna-Tobias-Klein barostat. Before performing the main simulations, a series of minimizations and short MD simulations were performed to relax the model system.

### Sample preparation

The protein stability was added to 2 ml screw-capped vials in phosphate buffer, at 25 °C and kept for 4 h to attain complete equilibrium after blending the solution. Similarly, the protein was dissolve in the mixture of 500 mM of osmolytes (sorbitol or trehalose) and phosphate buffer at 25 °C, and keep the solution for 4 h to attain complete equilibrium after blending the solution. The samples were treated at 6 mm distances from the plasma for 5, 10, and 20 min, at humidity of 40%, and were then incubated for 4 h at room temperature after plasma treatment. Three samples were treated for each condition to minimize the error.

### Statistical analysis

All of the values are represented by the mean ± S.D of the indicated number of replicates.

## Electronic supplementary material

Supporting Information
