# Supplementary material for: The protective action of osmolytes on the deleterious effects of gamma rays and atmospheric pressure plasma on protein conformational changes
Source: Sci Rep. 2017 Aug 18;7:8698. doi: 10.1038/s41598-017-08643-1 (PMC5562882; doi:10.1038/s41598-017-08643-1)
Supplement: Supplementary file 1 — Supporting Information [file 41598_2017_8643_MOESM1_ESM.pdf]

Supporting file

The protective action of osmolytes on the deleterious effects of gamma rays and atmospheric pressure plasma on protein conformational changes

Pankaj Attri[1,2], Minsup Kim[3], Thapanut Sarinont[4], Eun Ha Choi[1], Hyunwoong Seo[2], Art E. Cho[3], Kazunori Koga[2], and Masaharu Shiratani[2]

[1]Plasma Bioscience Research Center/Department of Electrical and Biological Physics, Kwangwoon University, Seoul 01897, Korea.
[2]Faculty of Information Science and Electrical Engineering, Kyushu University, Fukuoka, Japan.
[3]Department of Bioinformatics, Korea University, Sejong 02841, Korea.
[4]Graduate School of Information Science and Electrical Engineering, Kyushu University, Fukuoka, Japan.

Figure S1: Chemical denaturation profile of Myoglobin after treatment with different gamma rays absorbed dose such as 1136, 424 and 228 Gy (a) control Myoglobin, (b) Myoglobin with sorbitol and (c) Myoglobin with trehalose.

Figure S2: Chemical denaturation profile of Myoglobin protein after treatment with DBD plasma for different time intervals such as 20, 10 and 5 mins (a) control Myoglobin, (b) Myoglobin with sorbitol and (c) Myoglobin with trehalose.

Figure S3: Far-UV CD spectra for myoglobin at 222 nm after treatment with different gamma rays absorbed dose such as 1136, 424 and 228 Gy (a) control Myoglobin, (b) Myoglobin with sorbitol and (c) Myoglobin with trehalose.

Figure S4: Far-UV CD spectra for myoglobin at 222 nm after treatment with DBD plasma for different time intervals such as 20, 10 and 5 mins (a) control Myoglobin, (b) Myoglobin with sorbitol and (c) Myoglobin with trehalose.

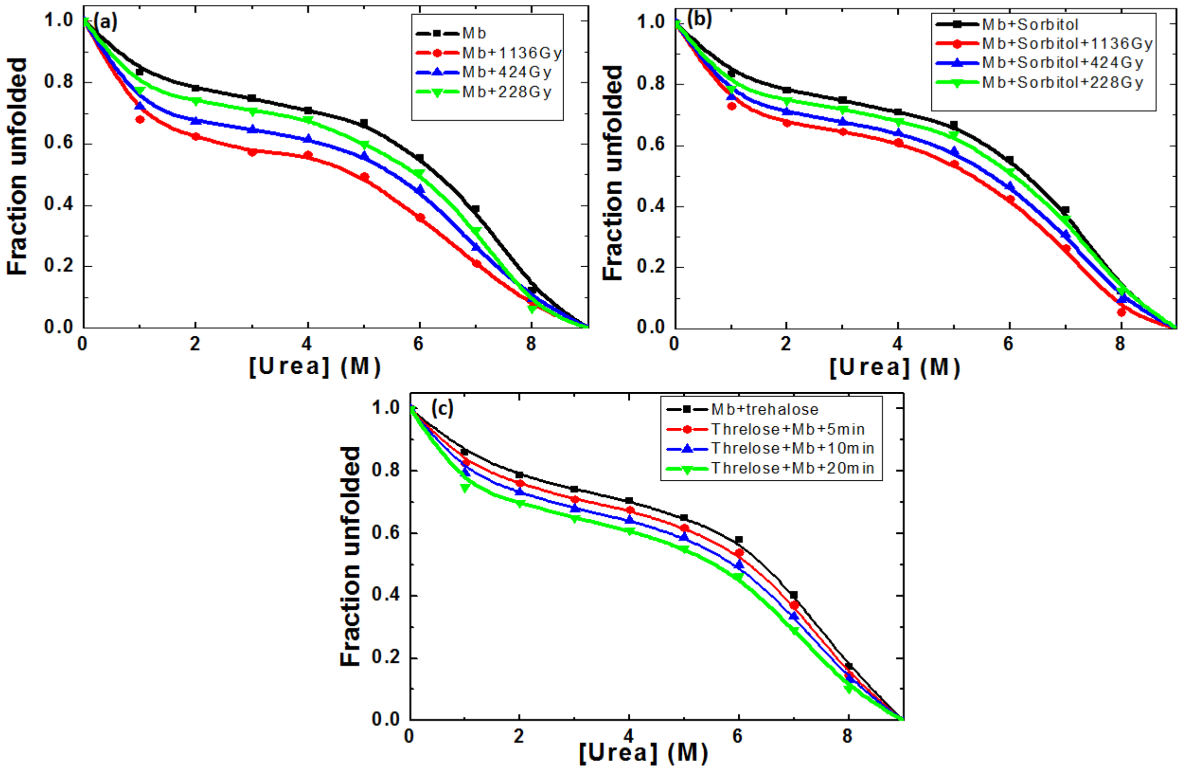

Figure S1

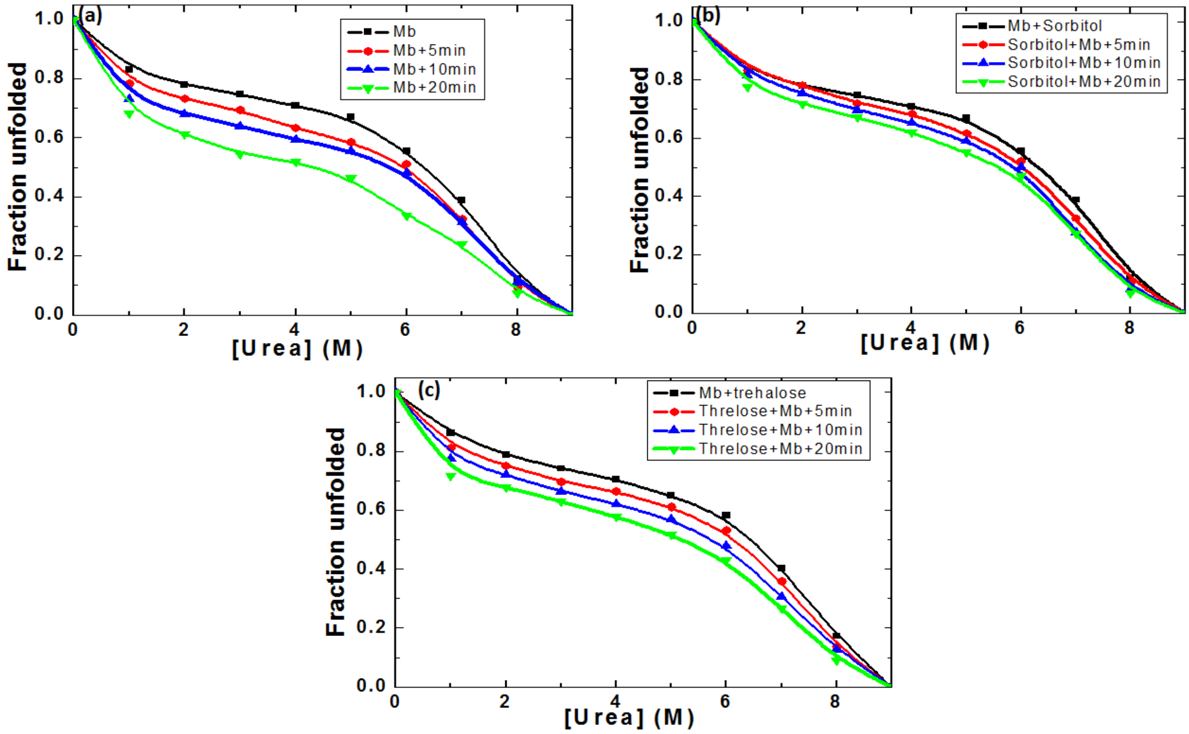

Figure S2

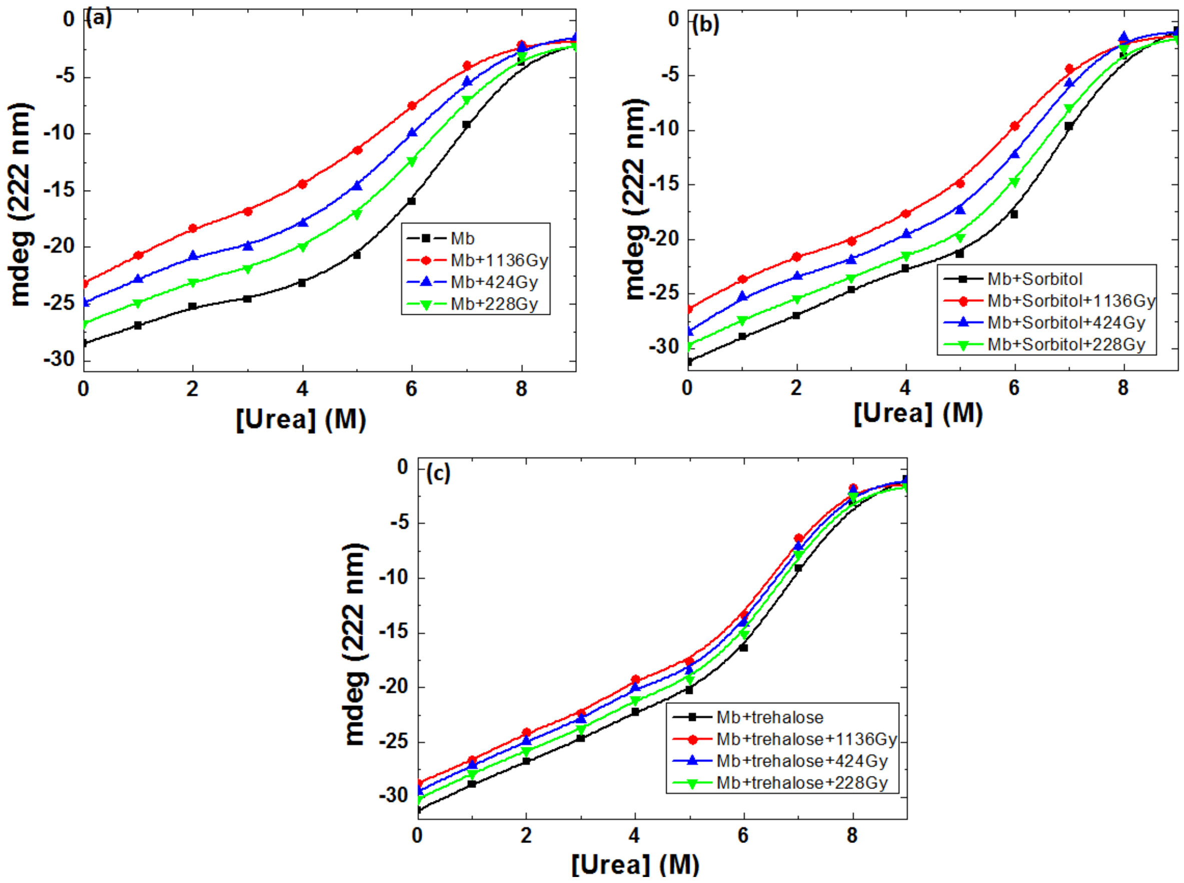
(a)
mdeg (222 nm)
[Urea] (M)
Mb
Mb+1136Gy
Mb+424Gy
Mb+228Gy
(b)
mdeg (222 nm)
[Urea] (M)
Mb+Sorbitol
Mb+Sorbitol+1136Gy
Mb+Sorbitol+424Gy
Mb+Sorbitol+228Gy
(c)
mdeg (222 nm)
[Urea] (M)
Mb+trehalose
Mb+trehalose+1136Gy
Mb+trehalose+424Gy
Mb+trehalose+228Gy

Figure S3

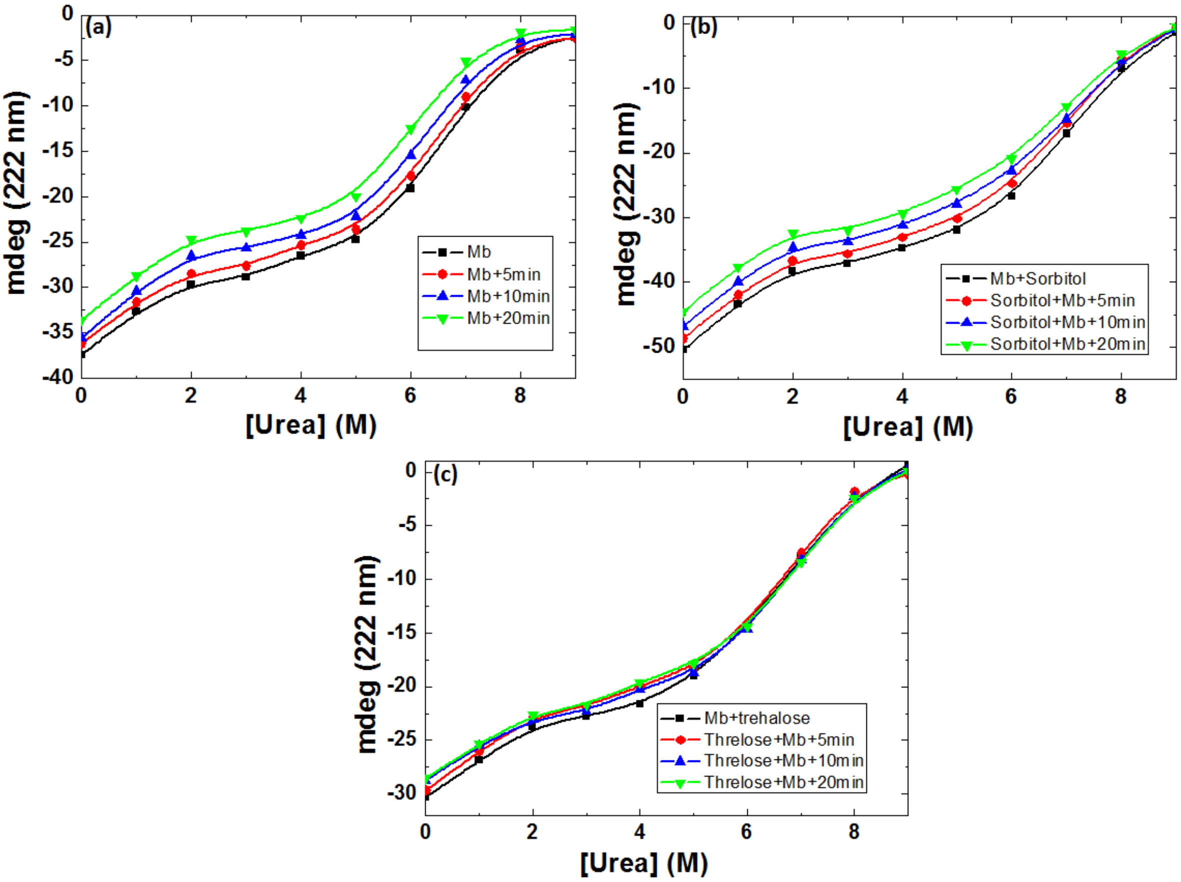

(a)
mdeg (222 nm)
[Urea] (M)
Mb
Mb+5min
Mb+10min
Mb+20min
(b)
mdeg (222 nm)
[Urea] (M)
Mb+Sorbitol
Sorbitol+Mb+5min
Sorbitol+Mb+10min
Sorbitol+Mb+20min
(c)
mdeg (222 nm)
[Urea] (M)
Mb+trehalose
Threlose+Mb+5min
Threlose+Mb+10min
Threlose+Mb+20min

Figure S4
